# Examining Virtual Delivery of Strength at Home for Intimate Partner Violence Perpetration

**DOI:** 10.3390/bs14121127

**Published:** 2024-11-25

**Authors:** Casey T. Taft, Justin K. Benzer, Megan Kopitsky, Suzannah K. Creech

**Affiliations:** 1 Behavioral Science Division, National Center for PTSD, VA Boston Healthcare System, 150 South Huntington Avenue, Boston, MA 02130, USA; megan.kopitsky@va.gov; 2 Boston University Chobanian and Avedisian School of Medicine, 72 East Concord Street, Boston, MA 02118, USA; 3 VA VISN 17 Center of Excellence for Research on Returning War Veterans and the Central Texas Veterans Healthcare System, 4800 Memorial Drive Building 93, Waco, TX 76711, USA; justin.benzer@austin.utexas.edu (J.K.B.); suzannah.creech@austin.utexas.edu (S.K.C.); 4 Dell Medical School, University of Texas at Austin, 1601 Trinity Street Building A, Austin, TX 78712, USA

**Keywords:** intimate partner violence, technology-delivered intervention, partner aggression, Strength at Home, implementation

## Abstract

This study examined the effectiveness of the virtual delivery of the Strength at Home (SAH) intervention program for intimate partner violence in a sample of 605 military veterans across 69 Veterans Affairs (VA) Medical Centers through a national implementation of the program. Outcome measures included physical IPV, psychological IPV, coercive control behaviors, post-traumatic stress disorder (PTSD) symptoms, and alcohol misuse. Significant pre-intervention to post-intervention reductions were found for all the outcomes, with similar effect size estimates relative to a prior investigation of in-person-delivered SAH through the same national VA implementation. Study findings suggest that the virtual delivery of SAH may be as effective as in-person delivery which has important implications for program access and impact.

## 1. Introduction

Intimate partner violence (IPV), specifically physical and psychological aggression toward an intimate partner, continues to be a public health crisis that affects millions of Americans each year [1]. In the United States, approximately one in four women and one in five men report the experience of IPV in their lifetime [2,3] and consequences range from extensive negative mental and physical health outcomes to death, including both homicide and suicide [4,5,6]. Physical, mental, sexual, and reproductive health consequences have been linked to IPV [7], and the impacts of IPV extend beyond the couple. Children exposed to IPV are at an increased risk for psychological, social, emotional, and behavioral problems, and are also more likely to engage in IPV later in life [8,9].

The modes of intervention for preventing family violence have changed since the lockdowns stemming from COVID-19, including a shift from the provision of in-person services to the use of virtual or telehealth modes of intervention [10,11,12]. While these forms of service delivery have been studied previously with respect to those who experience IPV [13,14], we are not aware of any published empirical investigation of virtual interventions for those who engage in IPV. The current study examined the effectiveness of the virtual delivery of an evidence-based intervention for IPV perpetration in a sample of military veterans.

Prior meta-analyses show that those referred to IPV intervention programs demonstrate only a 5% reduction in recidivism relative to untreated groups [15]. The studies of gold-standard IPV assessment methods using reports from survivors show a lack of significant reductions in IPV [16]. This lack of demonstrated intervention effectiveness is especially troubling considering that approximately half a million men and women are court mandated to more than 2500 of these programs each year [17,18]. Recent studies demonstrate that the Strength at Home (SAH) intervention for those who engage in IPV has shown promise in reducing IPV and other trauma-related problems such as post-traumatic stress disorder (PTSD) and alcohol use problems in military veterans [19,20] and civilians [21]. For all of these prior studies, however, the intervention was delivered in person. The intervention, which is the only such program promoted as “evidence-based” for veterans by the United States Department of Veterans Affairs, is currently primarily delivered virtually across more than 150 VA medical centers, and therefore, it is important to examine if this mode of delivery exhibits similar effect size reductions in the outcomes of interest relative to earlier work.

Anecdotally, providers report a number of benefits and drawbacks to delivering interventions for IPV perpetration virtually. As with the virtual delivery of interventions for a range of other problems [22], this mode may enhance attendance by lessening transportation and childcare barriers. Clients with extensive trauma histories in particular may also experience less anxiety participating from their home setting rather than an unfamiliar external setting with other participants who they do not know. It can be argued that with advances in technology, greater attendance can assist in developing a positive group environment, although there is also the sentiment among some that group cohesion may be better developed in person. Compliance with practice assignments may be more challenging with virtual delivery, as it becomes more difficult to hand out and collect group assignments. There are also often more distractions at home where the partners (survivors) of these clients may reside, as well as others, which has the potential to compromise privacy and safety. Other potential barriers may include limited internet access, low client digital literacy, and technical challenges in using such technology [13].

The recent reviews of interventions for those who experience IPV indicate the effectiveness of virtual delivery [13,14]. Cantor et al. [13] conducted a comparative effectiveness review of 17 studies using different methodologies to examine the effectiveness and harms of the telehealth delivery of interventions for women’s reproductive health and IPV services. Findings for IPV indicated similar results for telehealth versus usual care for repeat IPV, depression, post-traumatic stress disorder (PTSD), fear of partner, coercive control, self-efficacy, and safety behaviors. Emezue and colleagues [14] conducted a meta-analysis of 17 randomized controlled trials examining technology-based or digital interventions for depression, anxiety, PTSD outcomes, and victimization outcomes among the survivors of IPV. The pooled results indicated significant reductions in all the outcomes other than PTSD and sexual violence, suggesting the effectiveness of this modality for a number of problems.

This study examined the effectiveness of the virtual delivery of SAH to prevent and end IPV within the context of a program evaluation of a national implementation of the program across the Department of Veterans Affairs (VA) [19]. A prior study indicated that the in-person administration of this intervention within the VA was effective in reducing physical IPV, psychological IPV, and coercive control behaviors, as well as the veteran symptoms of PTSD and alcohol use problems [19]. The current study examined effect size reductions for these same outcomes for the veterans who received this intervention virtually following the onset of COVID-19 during a time in which in-person services were not available in VA settings. Significant reductions in all the outcomes of interest were expected, with similar effect size estimates for the participants receiving virtual SAH as has been reported in the previous Creech et al. [19] investigation.

## 2. Materials and Methods

The institutional review board at VA Boston evaluated the procedures and determined that this program evaluation of patient-level implementation outcomes did not meet the criteria for human research, thus exempting it from additional review by the board. To maintain patient privacy, individual patient data were anonymized and deidentified. This study is a nonrandomized assessment of implementation outcomes focused on quality improvement and not an experimental trial. As such, the study adheres to the reporting guidelines set forth by the revised Standards for Quality Improvement Reporting Excellence (SQUIRE) [23].

### 2.1. Participants

Patients in this study (*N* = 605) were mostly male veterans treated by VA clinicians participating in a national implementation of SAH across the VA healthcare system between March 2020 and September 2021. The veterans were aged 21 to 77 years, with 19% self-identifying as Black, 13% Hispanic, 62% non-Hispanic White, and 6% identifying as another race or ethnicity (including Alaska Native, Asian, Native American, Native Hawaiian or Pacific Islander, and multiracial). See Table 1 for the complete demographics. This sample consisted of veterans who were engaged with the criminal legal system due to IPV charges (62%). Data were derived from 69 VA medical centers across the country that offered SAH virtually to patients as a part of the national implementation. SAH is a trauma-informed group intervention based on a social information processing model of trauma and IPV, which asserts that those who engage in IPV possess social information processing deficits that impede one’s ability to accurately perceive social stimuli and encourage or enable aggressive behavior in response [24]. SAH promotes a positive group process and therapeutic relationship, and comprises 12 weekly two-hour group sessions that address enhancing motivation and accountability, the relationship between trauma and IPV, stress management skills, conflict management, and communication skills. 

Before joining a SAH group, all the patients underwent a baseline assessment and interview. During this process, they signed the necessary release of information forms and completed self-report measures related to IPV, PTSD symptoms, and alcohol misuse. It was not required that the patients have any primary diagnosis or a history of trauma to be eligible to receive SAH. Further, co-morbid mental health conditions did not exclude veterans from participation. The patients also disclosed their era of military service, gender, age, and race and ethnicity during the intake interview. Demographic data were gathered in order to provide perspective on how the program may have affected different populations of veterans.

SAH clinicians, who were VA employees primarily in the Social Work department, underwent a two-day workshop wherein they were taught how to facilitate the program. This workshop was led by program developers, or “regional trainers” trained by the program developers, and included role-play exercises and didactics. The trained clinicians were then required to attend weekly clinical consultations while facilitating their first two SAH cohorts. The groups typically consisted of 5–8 veterans and were co-facilitated by one to two SAH clinicians.

After the initial baseline assessment, the patients attended up to 12 weekly sessions of the SAH group. All the sessions were conducted using the same program that is delivered in person, though the participants were informed of additional group expectations regarding privacy (e.g., participating alone in a quiet place) and compliance (e.g., keeping cameras on during sessions). At the end of session 12, the same measures completed during the baseline assessment were re-administered to veterans, as well as a post-intervention satisfaction measure. Attendance in 9 out of the 12 sessions (75%) was required to attain program completion, and those who fulfilled this criterion received a certification of completion. To evaluate the program’s effectiveness, SAH clinicians submitted deidentified and anonymized scale scores and patient demographic information to the program evaluation staff. All the data were entered directly into the study database software by trained research assistants.

### 2.2. Study Measures

IPV was assessed using questions derived from the Centers for Disease Control and Prevention 2010 National Intimate Partner and Sexual Violence Survey (NIPSVS) [25]. The survey consisted of 30 items that examined four categories of IPV behavior: (1) psychological aggression (e.g., I acted very angry towards my partner in a way that seemed dangerous), (2) coercive control (e.g., I tried to keep my partner from seeing or talking to their family or friends), (3) reproductive control (e.g., I tried to get my partner pregnant when they did not want to get pregnant), and (4) physical aggression (e.g., I slapped my partner). Veterans reported if they engaged in behaviors from each of the four IPV categories in the past 3 months, both before and after intervention. The participants used a dichotomous scale to respond, with 0 indicating “no” and 1 indicating “yes” for each type of behavior. The prevalence of each type of IPV was calculated by summing the number of positive responses and converting the sum into a dichotomous variable to indicate whether each type of IPV was present or absent in the past 3 months. Summary scores were also calculated to reflect the total number of IPV types experienced (psychological, coercive control, reproductive control, and physical) with possible values ranging from 0 to 4.

The severity of post-traumatic stress disorder symptoms was assessed using the 20-item PTSD Checklist for the Diagnostic and Statistical Manual of Mental Disorders (Fifth Edition) (PCL-5) [26]. The PCL-5 is a clinical screening instrument that consists of questions attending to the 20 symptoms of PTSD as defined in the Diagnostic and Statistical Manual of Mental Disorders. The patients were asked to rate how frequently they were troubled by each symptom during the past month based on “a very stressful experience”. All the questions are scored on a 5-point Likert scale where 0 = not at all and 4 = extremely, with a total range of possible scores from 0 to 80. To calculate the severity of symptoms, the scores of individual items were summed, with higher scores indicating more severe symptoms. Sum scores at or above 33 have been associated with diagnostic levels of PTSD symptoms [27]. The PCL-5 has demonstrated high internal consistency, test–retest reliability, and convergent and divergent reliability with other measures of PTSD and trauma [27].

The Alcohol Use Disorders Identification Test (AUDIT) [28] was utilized to assess alcohol misuse in the previous year. The AUDIT is a 10-item, self-report measure that addresses the frequency and quantity of alcohol use, alcohol dependence, and problems caused by alcohol. A total AUDIT sum score was calculated by the intake clinician and then provided to the study site and used as a continuous score in analytical models. Possible scores range from 0 to 40 with higher scores indicating greater levels of alcohol misuse and a score of 8 or above being indicative of problematic or hazardous drinking [29]. The AUDIT has demonstrated adequate construct- and criterion-related validity and reliability [30].

### 2.3. Statistical Analysis

Data analysis involved comparing the pre-treatment and post-treatment IPV, PCL, and AUDIT scores. The sample consisted of all the patients who attended at least one virtual SAH group session and had non-missing IPV, PCL, and AUDIT scores. The majority of the missing data consisted of post-treatment outcomes. Regarding missing data, 169 individuals had complete data (28%; compared to 33% in Creech et al. [19]) and 378 participants had no post-treatment data (63%; compared to 51% in Creech et al. [19]). Multiple imputation was employed to estimate the missing post-treatment data by utilizing the pre-treatment scores, the number of treatment sessions attended, and demographic and lifetime IPV variables that demonstrated significant correlations with the observed scores and/or the absence of data. Multiple imputation was performed using SAS 9.4 applying the Markov chain Monte Carlo (MCMC) method for the AUDIT and PCL measures, and the fully conditional specification (FCS) method for the dichotomous IPV measures. We set the parameters for 20 imputations and 100 burn-in iterations. The difference between Time 1 and Time 2 measures was assessed using the McNemar chi-square tests for binary variables and one-sided t-tests for interval and count variables. Effect sizes were calculated as odds ratios for binary variables and Hedge’s G for interval and count variables. Significance was established using the Benjamani–Hochberg method with a false discovery rate of 5%.

## 3. Results

A total of 605 veterans completed an intake for the SAH program. Table 2 provides the changes in IPV prevalence. There were significant reductions from pre- to post-treatment in the proportion of the sample who reported physical IPV [percent change, −0.20; 95% CI, −0.26 to −0.14], psychological IPV [percent change, −0.27; 95% CI, −0.34 to −0.20], coercive control behaviors [percent change, −0.19; 95% CI −0.27 to −0.12], and the presence of any IPV [percent change −0.27; 95% CI, −0.34 to −0.19]. The frequency of reproductive control was low (<2% at Time 1) and no changes were observed.

Table 3 presents the changes in the sum of the number of IPV subtypes, PTSD symptoms, and alcohol misuse. Significant improvements were observed for all three outcomes. Effect sizes were small for PTSD symptoms (mean change, −9.99; 95% CI, 2.84 to 17.15; Hedges G = 0.32) and alcohol misuse (non-significant mean change; Hedges G = 0.18) and medium for the number of IPV subtypes reported (mean change 0.64; 95% CI, 0.50 to 0.79; Hedges G = 0.65).

## 4. Discussion

Consistent with expectations, this examination of SAH delivered virtually demonstrated significant reductions in all the outcomes of interest including physical IPV, psychological IPV, coercive control, PTSD symptoms, and alcohol misuse. Also as expected, similar effect size estimates were found in this study for all the outcomes relative to a prior investigation of in-person-delivered SAH through the same national implementation of the program in the VA [19]. While we were not able to make direct statistical comparisons between effect sizes in these two studies, in all the cases, the magnitude of effects was similar and slightly larger in the current study of virtual SAH delivery.

The results contribute to a growing evidence base demonstrating that SAH is an effective intervention for preventing and ending IPV and reducing other trauma-related problems such as PTSD symptoms and alcohol use problems [19,20,21,31,32]. The program now has demonstrated effectiveness for veterans and civilians, and when administered in person or virtually. It cannot be assumed that other IPV intervention programs can similarly be equally effective across different modes of intervention. It may be that interventions such as SAH that are trauma-informed and that emphasize a therapeutic group process and client–provider working alliance could better overcome some challenges encountered virtually for creating a positive group environment than programs guided by a different philosophy and theoretical framework.

While research is needed to evaluate the virtual delivery of other IPV programs to determine the generalizability of the current findings, these results are generally promising for the virtual delivery of IPV intervention programs, as has been shown for interventions for IPV survivors [13,14]. This may have important implications considering potential barriers to in-person attendance, such as transportation and financial difficulties, childcare barriers, and mental health issues such as social anxiety and post-traumatic stress disorder. Prior to lockdowns stemming from COVID-19, it was commonly believed that IPV interventions could not be safely and effectively delivered virtually. The current study provides some initial evidence that the virtual administration of IPV intervention may be at least as effective as in-person delivery.

The virtual delivery of IPV intervention may improve access in other ways. It is common for courts in some jurisdictions to not have a local IPV intervention program to refer clients. Reliance on in-person attendance requires that the program or provider for IPV intervention be in close proximity. Evidence for virtual IPV intervention, such as that shown in this study, suggests that a program or provider need not be local to the individual client, which allows for the possibility of attending programs at a greater distance, which should allow for greater coverage for abusive individuals and potentially greater reach and impact of intervention programs to prevent and end IPV.

Positive results for virtual IPV intervention delivery may also indicate a necessity for programs to review their policies about the mode of delivery, and for states to revisit IPV intervention practice guidelines. It is common for programs and state standards to only allow for virtual delivery under special circumstances. If virtual IPV intervention is indeed as effective as in-person delivery, practice guidelines should not prioritize in-person delivery. IPV practice guidelines should always strive to be evidence-based rather than based on assumptions and clinical lore [33].

There are caveats to consider when interpreting the study results. Since the participants were not randomized between delivery systems and there was no control condition, we cannot make definitive statements about effectiveness, and thus, the current findings should be viewed as preliminary. Future studies are needed to compare different modes of program delivery directly in order to determine the most effective method, and perhaps for whom different modes of delivery work best. Additional technologies, such as the use of applications (apps), or rapidly developing artificial intelligence systems, may also be studied in future research to examine their additional benefits. It would also be important to examine the virtual delivery of SAH and other IPV interventions in civilian and other samples that may differ with respect to education level and access to technology.

Despite its limitations, this study provides additional evidence for the effectiveness of SAH and an early step in demonstrating that virtual intervention for those who engage in IPV can be effective. Considering that IPV interventions have been shown to be relatively ineffective, it is critically important that researchers continue to evaluate innovative interventions and delivery methods to maximally address this serious public health issue.

## Figures and Tables

**Table 1:** Veteran demographic and completion data.

	Full Sample *N* = 605		Subset with Post-Treatment Data ^a^ *N* = 227	
Sample Characteristic	*N*/Mean	%/SD	*N*/Mean	%/SD
Age	44.36	12.40	44.67	12.53
Sex				
Male	569	94%	217	96%
Female	27	4%	8	4%
Missing	9	1%	2	<1%
Court Involved				
Yes	376	62%	170	75%
No	215	36%	55	24%
Missing	14	2%	2	<1%
Race				
Alaska Native or Native American	5	1%	1	<1%
Asian	5	1%	1	<1%
Black	115	19%	39	17%
Native Hawaiian or Pacific Islander	2	<1%	2	<1%
White Hispanic	46	8%	20	1%
White Non-Hispanic	377	62%	135	59%
Multiple Races	11	2%	6	3%
Missing	14	2%	14	6%
Ethnicity				
Hispanic	79	13%	37	16%
Non-Hispanic	512	85%	184	81%
Missing	17	3%	6	2%
Completed ≥ 9 sessions				
Yes	433	72%	215	95%
No	172	28%	12	5%

Table note: ^a^ refers to the patients who completed at least one post-treatment outcome measure.

**Table 2:** Effect of SAH on IPV prevalence.

IPV	Time 1 *N* (%)	Time 2 *N* (%)	Odds Ratio	Diff	95% Confidence Interval	
Physical IPV	161 (27%)	40 (7%)	4.22	−0.20 *	−0.26	−0.14
Psychological IPV	269 (44%)	106 (17%)	4.10	−0.27 *	−0.34	−0.20
Coercive Control	202 (33%)	87 (14%)	4.13	−0.19 *	−0.27	−0.12
Any IPV	324 (54%)	161 (27%)	3.45	−0.27 *	−0.34	−0.19

Note: * significance of the difference scores evaluated using the Benjamani–Hochberg (B-H) score. There was no change in reproductive control (*n* = 12).

**Table 3:** Effect of SAH on number of IPV subtypes reported, PTSD symptoms, and alcohol misuse.

Variable	Time 1	Time 2	Hedges G	Diff	95% Confidence Interval	
IPV Subtypes	1.06	0.42	0.65	0.64 *	0.50	0.79
PTSD Symptoms	36.62	26.62	0.32	9.99 *	2.84	17.15
Alcohol Misuse	6.51	3.98	0.18	2.53	−0.07	5.72

Note: *N* = 605; * significance of the difference scores evaluated using the Benjamani–Hochberg (B-H) score.

## Data Availability

The datasets presented in this article are not readily available due to legal restrictions and institutional policies. The minimal dataset underlying the results described in the manuscript may be made available upon request from the corresponding author pending appropriate institutional approval.
